# Obstructed Left Paraduodenal Hernia: A Rare Cause of Acute Abdomen in a COVID-19 Patient

**DOI:** 10.7759/cureus.16265

**Published:** 2021-07-08

**Authors:** Sanoop K Zachariah, Anil K P, Reji Jayadas, Maya Devi

**Affiliations:** 1 General and Minimal Access Surgery, Kerala Institute of Medical Sciences-KIMSHEALTH, Trivandrum, IND

**Keywords:** paraduodenal hernia, covid -19, left paraduodenal hernia, internal hernia, intestinal obstruction, hernia, acute abdomen, ct scan, bowel obstruction, treitz hernia

## Abstract

A paraduodenal hernia (PDH) is a rare type of internal hernia, which results from anomalous rotation and reduction of the midgut loop in the embryo. The diagnosis is often difficult due to nonspecific symptoms. The mortality from an acute internal hernia can be close to 50% when the diagnosis and definitive surgical treatment are delayed. Here we present a rare case of obstructed left paraduodenal hernia (LPDH) in a COVID-positive patient. This is probably the earliest report of acute mechanical intestinal obstruction due to LPDH in a COVID-positive patient.

## Introduction

Paraduodenal hernias (PDH) are rare congenital anomalies of the gut resulting from defective rotation and fusion in the embryo leading to the bowel being entrapped within potential spaces or sacs that are lined by peritoneum [1]. They constitute 50% of congenital internal hernias. They are three times more frequent in males and usually present around the third or fourth decades of life with the average age of diagnosis at 38.5 years. In about 75% of the cases, PDH occurs to the left of the midline in the paraduodenal fossa of Landzert and is referred to as left paraduodenal hernia (LPDH), while in 25% of the cases, it occurs through the fossa of Waldeyer and is termed right paraduodenal hernia (RPDH) [2]. Asymptomatic PDH may be incidentally discovered at laparotomy for some other condition. Symptomatic PDH usually present with vague nonspecific symptoms and are therefore difficult to diagnose and hence the definitive treatment may be delayed. The reported mortality is as high as 50% in untreated cases. Therefore, early diagnosis and treatment are essential in limiting morbidity and mortality. To the best of our knowledge, an LPDH presenting as an acute abdominal surgical emergency in a COVID-positive patient has not yet been reported in the literature.

## Case presentation

A 41-year-old male presented to the emergency room with acute onset colicky abdominal pain for two days, associated with absolute constipation. He described having experienced postprandial bloating and abdominal discomfort for one week. The patient was a fisherman and resident from a designated COVID containment zone. He had no history of chronic abdominal pain, previous abdominal surgeries, or comorbidities. No history was suggestive of coronavirus disease 2019 (COVID-19) infection. Abdominal examination revealed tenderness in the umbilical, left hypochondrium, and left iliac regions. Bowel sounds were hypoperistaltic. He was afebrile, with normal vital signs. Blood analysis indicated increased WBC counts (12000/cumm) with leucocytosis. Ultrasonography of the abdomen was inconclusive. The COVID test reverse transcription-polymerase chain reaction (RT-PCR) assay for severe acute respiratory syndrome (SARS)-associated coronavirus was negative. 

**Figure 1:**
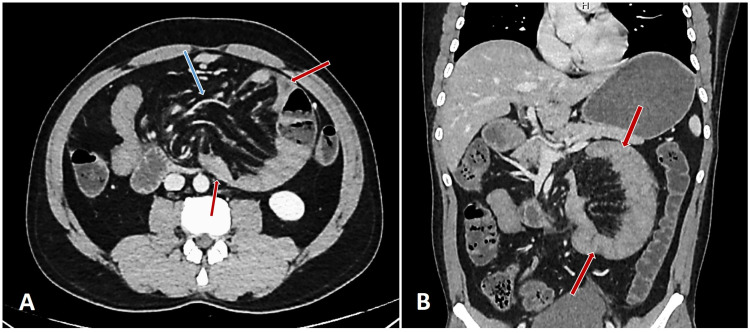
Contrast-enhanced computed tomography (CECT) findings Axial (A) and coronal (B) views of CECT showing typical findings, left paraduodenal hernia (LPDH) namely herniation of bowel loops through an orifice  (blue arrow) and clustered small bowel loops to the left upper quadrant in the anterior pararenal space forming a C- shaped closed loop (red arrows).

A contrast-enhanced computed tomography (CECT) scan of the abdomen suggested the possibility of LPDH with features of intestinal obstruction and this clinched the diagnosis (Figure 1).

**Figure 2:**
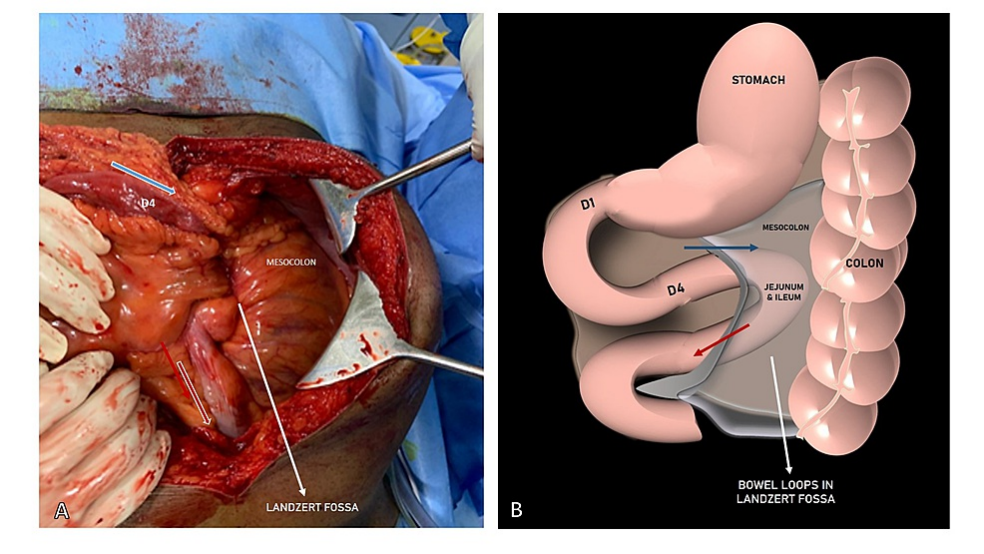
Intraoperative image of left paraduodenal hernia (LPDH) (A) Shows the afferent limb beyond the fourth part of the duodenum depicted by the blue arrow and the efferent limb (red arrow) exiting from the Landzert fossa; (B) schematic representation of the LPDH; D1 is the first part of the duodenum and D4 is the fourth part of the duodenum.

During emergency exploratory laparotomy, the majority of the small bowel was found to be herniated into the lesser sac. The sac-like mass contained dilated jejunal and proximal ileal loops alongside the fourth part of the duodenum through an internal opening, namely the paraduodenal fossa of Landzert, near the ligament of Treitz, below the transverse mesocolon (Figure 2).

**Figure 3:**
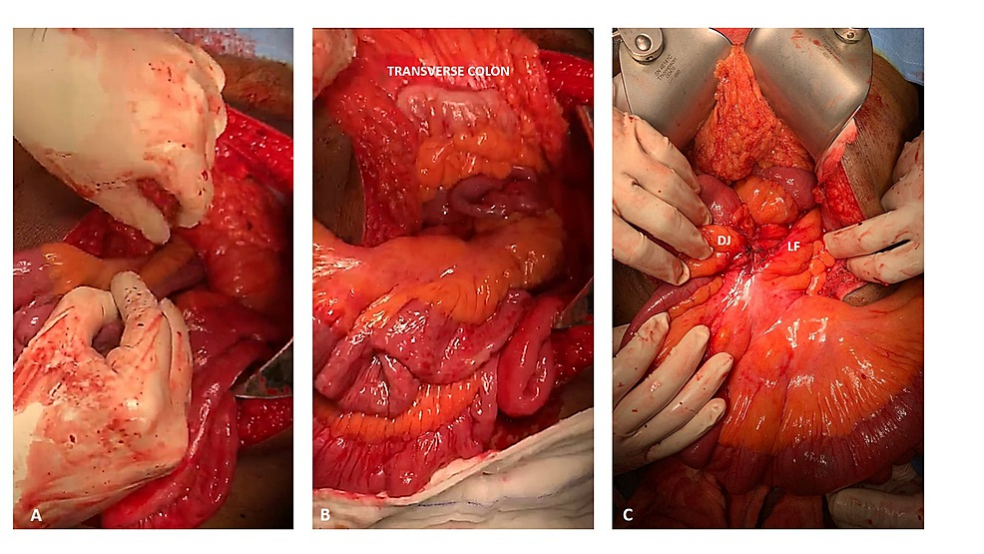
Intraoperative image - reduction of the left paraduodenal hernia (LPDH) (A) manual reduction of the bowel loops into the peritoneal cavity; (B) the reduced bowel loops lying below the transverse mesocolon; (C) the bowel loops after complete reduction showing the duodenal jejunal junction (DJ) on the right and the Landzert fossa on the left of the DJ.

About two feet of the intestinal loops comprising of the jejunum and proximal ileum were meticulously reduced into the peritoneal cavity. Although the intestinal loops appeared dusky initially, the congestion slowly waned off after reduction, and the normal colour with good peristalsis was restored, confirming the viability of the bowel. The rest of the solid and hollow organs appeared normal (Figure 3).

On the second postoperative day, the patient developed high-grade fever. His abdomen was soft and he was tolerating oral fluids. Bowel movements were normal. Based on a high index of clinical suspicion, the RT-PCR test was repeated, and it turned out to be positive. The dimer value was 2388 ng/ml by enzyme-linked immunofluorescence assay (ELFA).

The patient was shifted to the COVID care unit. The fever lasted for three days and was treated according to the hospital's COVID treatment protocol. The patient made an uncomplicated recovery and was discharged after two weeks when the COVID test result was negative. The patient is on regular follow up for 10 months and is doing well.

## Discussion

An LPDH is defined as an abnormal protrusion of the bowel into the Landzert fossa. This fossa is a space that typically gets obliterated around five to ten weeks of embryonic life, when the left mesocolon ascending left colic artery and the inferior mesenteric vein (IMV), fuses with the retroperitoneum. This occurs at the same time when the small bowel is completing the 270-degree anti-clockwise rotation about the superior mesenteric artery (SMA) [3-5].

In about 1% to 2% of the people, it is thought that bowel invagination into the avascular plane posterior to vessels behind the left mesocolon, predisposes to the formation of the fossa. Therefore, the anterior border of this orifice, the IMV, and the left colic artery are shifted or transposed anteriorly, and the left colon mesentery (mesocolon) forms the anterior wall of this hernia.

The afferent limb is generally formed by the jejunum close to the fourth portion of the duodenum, and the efferent limb can go as far as the ileum if extensive herniation occurs [6,7]. Awareness of the vascular anterior border of the aperture during repair of an LPDH is important.

The clinical features are usually nonspecific with a history of chronic intermittent, self-limiting postprandial abdominal pain with an inconclusive clinical workup. Up to 70% of patients give a history of chronicity of abdominal pain. The lifetime risk of strangulation and intestinal infarction is more than 50% [4].

CECT scan of the abdomen can depict pathognomonic findings and can probably be considered the gold standard investigation in PDH. CT appearance of LPDH includes clustering or bunching up of small bowel in the left side of the upper abdomen, into a closed-loop forming a ‘C’ or ‘U’ shaped mass like sacculation with the displacement of the stomach, transverse colon, and the duodenal-jejunal junction [8]. 

Our patient did not have chronic abdominal pain and he had experienced vague postprandial abdominal symptoms only for one week. Moreover, the patient was initially negative for COVID infection and this was probably due to testing in the initial latent phase of the infection. It is well known by now that coronaviruses commonly cause respiratory and/or enteric infections [9,10]. In severely affected COVID-19 patients, the incidence of gastrointestinal complications is high as 73.8%, including the occurrence of gastrointestinal ischemia (3.8%) [11]. Pathophysiological hypothesis includes microvascular thrombotic events and viral enteroneuropathy.

The commonly reported gastrointestinal manifestations in COVID patients include acute liver injury and elevated transaminases (66.6%), acute acalculous cholecystitis [12], acute pancreatitis, Ileus, feeding intolerance, acute colonic pseudo-obstruction [13], and mesenteric ischemia. All these are usually manifested in critically ill patients. The exact reasons are still being evaluated. There are reports of the presence of SARS coronavirus-2 (SARS-CoV-2) RNA from stool, bile, and pancreatic fluids in COVID-19 patients [14-16]. Intestinal obstruction in the form of acute colonic pseudo-obstruction or adynamic obstruction has been reported as a discrete clinical syndrome in critically ill COVID patients characterised by severe gaseous distension of the abdomen similar to Ogilvie's syndrome.

Our patient presented with definitive evidence of mechanical intestinal obstruction. It can be speculated that the COVID-19, to be a concomitant infection or the presence of the virus in the gastrointestinal system itself would have predisposed to intestinal distension due to viral enteroneuropathy, resulting in the occurrence of mechanical obstruction in an otherwise asymptomatic patient. Luckily there were no serious gastrointestinal ischaemic complications as the LPDH was diagnosed in time. As stools samples were not studied for SARS-CoV-2 RNA, it is uncertain if the gastrointestinal system was affected by COVID-19. 

To the best of our knowledge, this is probably the first report of acute intestinal mechanical obstruction due to a PDH in a COVID-positive patient. Our previous experience with PDH probably helped us in surgical management [17]. Adequate protection is necessary for the operating team even if the COVID test is negative. A high index of clinical suspicion is necessary for diagnosing PDH and a CT scan should be ordered, if in doubt. 

## Conclusions

Internal hernias are rare causes of acute intestinal obstruction and the diagnosis requires a high index of clinical suspicion. Such rare causes of an acute abdomen should not be overlooked even in COVID-positive patients. CT scan is the diagnostic modality of choice in PDHs. Timely recognition and surgical intervention can improve the outcome.
